# Safety of Low Dose Intravenous Cangrelor in Acute Ischemic Stroke: A Case Series

**DOI:** 10.3389/fneur.2021.636682

**Published:** 2021-06-04

**Authors:** Hisham Salahuddin, Giana Dawod, Syed F. Zaidi, Julie Shawver, Richard Burgess, Mouhammad A. Jumaa

**Affiliations:** ^1^ProMedica Neurosciences Center, Toledo, OH, United States; ^2^Department of Neurology, College of Medicine and Life Sciences, University of Toledo, Toledo, OH, United States; ^3^Department of Neurology, Antelope Valley Hospital, Lancaster, CA, United States

**Keywords:** thrombectomy, ischemic stroke, cangrelor, antiplatelet therapy, stent, tandem occlusion

## Abstract

**Background:** Neurointerventional procedures in acute ischemic stroke often require immediate antiplatelet therapy in the cases of acute stenting and occasionally re-occluding vessels. Intravenous cangrelor is a P2Y12 receptor antagonist with short onset and quick offset. The study objective was to evaluate the safety and efficacy of intravenous cangrelor in patients with acute ischemic stroke requiring urgent antiplatelet effect.

**Methods:** Patients who received intravenous cangrelor intra-procedurally during acute ischemic stroke treatment were identified from a prospectively collected database. Cangrelor was administered as a bolus of 15 mcg/kg, followed by an infusion rate of 2 mcg/kg/min. A historical control group consisting of anterior circulation tandem occlusions was used to compare to patients with similar lesions who received intravenous cangrelor. Outcomes of interest included in-stent thrombosis, thromboembolic complications, intracranial hemorrhage, and functional outcomes at 90 days.

**Results:** Twelve patients received intravenous cangrelor for acute ischemic stroke between October 2018 and April 2020 at a comprehensive stroke center. Eleven patients had intra or extracranial stenting performed, which included two posterior circulation lesions. No cases of symptomatic intracranial hemorrhage were reported. At 90 day follow-up, two patients had died and 10 had a good functional outcome. Patients with anterior circulation tandem occlusions who received cangrelor and those who received dual antiplatelets orally had similar radiographic and clinical outcomes.

**Conclusion:** Low dose intravenous cangrelor is similar in safety and efficacy to oral antiplatelets in acute ischemic stroke in a small case series. Larger prospective studies on the efficacy, safety, and effect on procedure times of intravenous cangrelor in neurointervention are warranted.

## Introduction

Mechanical thrombectomy for acute ischemic stroke often requires immediate antiplatelet therapy when underlying intracranial atherosclerotic disease is encountered, and this frequently needs to be weighed against the risk of bleeding. Current American Heart Association guidelines allow for consideration of antiplatelet therapy within 24 h of IV tPA administration when it provides substantial benefit or withholding such treatment is known to cause substantial risk (Level IA) (1). Administration of antiplatelet therapy for acute intracranial or extracranial stenting is often done with incomplete information such as final infarct volume, previous systemic bleeding complications, medication intolerances, and potential need for decompressive cranial surgery. Furthermore, to avoid the risk of aspiration due to oral administration of antiplatelet medications, emergent placement of a nasogastric tube before or during mechanical thrombectomy is frequently required, often after administration of intravenous tPA. Clopidogrel is a commonly used antiplatelet therapy in acute ischemic stroke, has a peak onset of ~2 h after a loading dose, may be affected by underlying co-morbidities, may interact with other medications, and has an unpredictable antiplatelet effect in patients with CYP genetic polymorphisms (2).

Intravenous cangrelor is a potent, reversible, non-thienopyridine ADP analog that provides adequate platelet inhibition within 3–6 min and has a short half-life. It does not require hepatic activation, can be infused through a peripheral intravenous line, and its antiplatelet activity can be accurately assessed by the VerifyNow PRU assay (3). Given the predictable pharmacokinetics in patients with a wide variety of underlying co-morbidities, it provides an important solution for acute neurointerventions. Randomized clinical trials in interventional cardiology have found that intravenous cangrelor may be a useful and cost-effective substitute for currently used oral antiplatelets (4–6). Given the differences in patient characteristics, clinical outcomes, and treatment considerations from cardiac patients, we sought to determine the safety and efficacy of using low dose cangrelor infusions in patients with acute ischemic stroke.

## Materials and Methods

After institutional board review approval, we reviewed consecutive patients in our prospectively collected acute ischemic stroke database for patients over 18 years who presented between October 2018 and April 2020 and received intravenous cangrelor within 6 h of hospital admission and underwent mechanical thrombectomy. We compared this group with consecutive patients from the same database who presented with anterior circulation tandem occlusions based on digital subtraction imaging who underwent mechanical thrombectomy in the previous 4 years (October 2014–October 2019) prior to the availability of intravenous cangrelor at our comprehensive stroke center. Exclusion criteria included patients who were already on dual antiplatelet therapy.

Baseline demographics, time metrics, neurointerventional procedural details, and follow-up neuroimaging were abstracted from our database. We retrospectively evaluated ischemic core volume on post-procedural MRI scans (or CT brain if not available) using the ABC/2 method (7). Follow-up mRS scores were obtained by in-person visits with a stroke physician at 90 days.

The primary study outcome was intracranial hemorrhage within 24 h of starting cangrelor infusion. Secondary outcomes included discharge disposition, disability at 90 days as assessed by the modified Rankin scale, worsening hemorrhage within 36 h of cangrelor infusion or any hemorrhage requiring discontinuation of intravenous cangrelor, and recurrent transient ischemic attack or ischemic stroke within 30 days.

### Treatment and Cangrelor Administration Protocol

Patients were selected for mechanical thrombectomy based on CT angiogram if they presented within 6 h of the last known normal, and CT perfusion if they presented between 6 and 24 h of the last known normal.

Patients requiring acute antiplatelet therapy regardless of intravenous tPA infusion were administered intravenous cangrelor in the neuroangiography suite immediately prior to stenting. Carotid artery stenting was always performed after recanalization of the intracranial occlusion. A bolus of 15 mcg/kg was administered over 2 min, followed by an infusion rate of 2 mcg/kg/min. We selected a 15 mcg/kg bolus followed by 2 mcg/kg/min dosing schedule for cangrelor given that it provides similar amounts of platelet aggregation and time to recovery of platelet function as the higher dose (8). Decision regarding acute stenting was left to the discretion of the neurointerventionalists. When acute stenting was required, cangrelor bolus was completed prior to stent placement. Assessment of P2Y12 levels was performed at the discretion of the treating provider. Patients were maintained on intravenous cangrelor for 12–24 h and transitioned to dual oral antiplatelet therapy after post-thrombectomy neuroimaging. Aspirin was administered prior to discontinuation of intravenous cangrelor. For patients who were unable to swallow, oral antiplatelets were administered through a nasogastric tube. If patients were transitioned to oral ticagrelor, this was administered immediately prior to cangrelor infusion discontinuation. If clopidogrel was chosen as the second antiplatelet, it was administered 15 min after discontinuing the intravenous cangrelor infusion. Choice of oral antiplatelet transition from cangrelor was left to the discretion of the treating neurointerventionalist.

### Statistical Analysis

Collected data were exported from Microsoft Excel into statistical software “R: A language and environment for statistical computing; EZR version 1.32” (Saitama Medical Center, Jichi Medical University, Saitama, Japan). Continuous and categorical variables are presented as mean (standard deviation), median (interquartile range), and percentages where appropriate. Continuous variables were analyzed with the Student *t*-test or Mann-Whitney test where appropriate, and categorical data were analyzed using the Fisher exact test. A *p*-value of ≤ 0.05 was considered significant. All analyses were two-tailed and the level of significance was set at 0.05.

## Results

Between October 2018 and April 2020, 12 patients received intravenous cangrelor during neurointerventional procedures in the setting of acute ischemic stroke (see Table 1). There were five (41.7%) women, mean age of the cohort was 67.9 ± 12, and median ASPECT/pc-ASPECT score was 9.5 (IQR 9–10). Median NIHSS on presentation was 15 (13–21), seven (58.3%) patients received intravenous tPA, and all patients achieved successful reperfusion (TICI 2b or greater) (Table 2 describes individual characteristic & treatment variables).

**Table 1:** Baseline demographics and treatment variables for patients who received intravenous cangrelor for acute ischemic stroke.

	**Cangrelor (*n* = 12)**
**Age**	67.9 ± 12
**Female**	5 (41.7)
**Hypertension**	10 (83.3)
**Type 2 diabetes mellitus**	4 (33.3)
HbA1c	6.5 ± 1.2
**Atrial fibrillation**	2 (16.7)
**Hyperlipidemia**	8 (66.7)
LDL (mean ± SD)	82.3 ± 24.3
**Smoking**	4 (33.3)
**CAD**	5 (41.7)
**NIHSS** (median; IQR)	15 (13–21)
**IV-tPA**	7 (58.3)
**ASPECTS / pc-ASPECTS** (median; IQR)	9.5 (9–10)
**Door to reperfusion** (min) (median; IQR)	84 (54–137)
**Onset to reperfusion** (min) (median; IQR)	252 (149–306)
**Time to follow-up scan** (min) (median; IQR)	680 (300–1,080)
**Procedure time (min)**	60 (48.5–133)
**Location of occlusion**	
MCA	6 (50)
M2 MCA	3 (25)
ICA terminus	1 (8.3)
Basilar artery	2 (16.7)
Tandem	10 (83.3)
**Successful reperfusion**	12 (100)
**Good clinical outcome**	10 (83.3)
**Mortality**	2 (16.7)
**sICH**	0
**Hemorrhagic transformation score**	
0	10 (83.3)
Hemorrhagic transformation type 1	2 (16.7)
Hemorrhagic transformation type 2	0
Parenchymal hematoma	0
**Duration on cangrelor (min; median)**	1,112 (995–1,426)
**Stent location**	
Proximal ICA	9 (75)
Basilar artery	1 (8.3)
Vertebral artery	1 (8.3)
**Stent patency**	11/11 (100%)
**Antiplatelet transition**	
Ticagrelor	4 (33.3)
Clopidogrel	8 (66.7)

^*^CAD, coronary artery disease; NIHSS, National Institute of Health Stroke Scale; MCA, middle cerebral artery; M2, M2 division of middle cerebral artery; ICA-T, internal carotid artery terminus; pc-ASPECT, posterior circulation Alberta Stroke Program Early CT Score; Successful RP, successful reperfusion; sICH, symptomatic intracranial hemorrhage; HT, hemorrhagic transformation score.

^**^Continuous variables represented as mean ± standard deviation or median (interquartile range).

**Table 2:** Baseline characteristics and treatment variables of patients receiving intravenous cangrelor for acute ischemic stroke.

**Subject**	**Age**	**Comorbidities**	**Baseline NIHSS**	**Site of occlusion**	**Tandem occlusion**	**Acute stenting**	**IV- tPA**	**HT**	Transitioned to:^**^	**mRS at 3 months**
1	60s	DM, HPL	11	R M2 tandem	Y	Y	N	N	Clopidogrel 300 + ASA 325 mg	2
2	70s	DM, HPL, HTN, CAD	14	R M2 tandem	Y	Y	N	N	Clopidogrel 600 + ASA 325 mg	2
3	40s	HTN	20	Basilar + R V4 occlusions	N	Y	Y	N	Clopidogrel 300 + ASA 325 mg	1
4	50s	HTN, HPL	12	R M1 tandem	Y	Y	Y	N	Clopidogrel 600 + ASA 325 mg	1
5	80s	DM, HPL, HTN, CAD, AF	27	L M2 tandem	Y	Y	Y	N	Ticagrelor 135 mg + ASA 325 mg	6
6	70s	-	23	Basilar occlusion	N	Y	N	N	Ticagrelor 180 mg + ASA 81 mg	6
7	60s	HTN, HPL	15	R M1 tandem	Y	Y	N	N	Clopidogrel 600 mg + ASA 81 mg	3
8	70s	HPL, HTN, CAD, AF	15	L ICA-T tandem	Y	Y	Y	HT1	Clopidogrel 300 + ASA 81 mg	2
9	60s	HTN, HPL, CAD	8	L M1 tandem	Y	Y	Y	HT1	Ticagrelor 90 mg + ASA 325 mg	1
10	60s	HTN	12	R M1 tandem	Y	Y	Y	N	Clopidogrel 300 + ASA 325 mg	0
11	70s	DM, HTN, smoking	25	L MCA tandem	Y	Y	Y	N	Clopidogrel 600 + ASA 81 mg	0
12	80s	HTN, HPL, CAD	13	R MCA occlusion	N	N	N	N	Ticagrelor 135 mg + ASA 81	2

^*^Y, yes; N, no; CAD, coronary artery disease; DM, diabetes mellitus; HTN, hypertension; AF, atrial fibrillation; HPL, hyperlipidemia; M1, M1 segment of MCA; M2, M2 segment of MCA; ICA-T, internal carotid artery terminus; NIHSS, NIH Stroke Scale; HT, hemorrhagic transformation (9); mRS, Modified Rankin Scale.

** *Maintenance doses were: aspirin 81 mg QD, ticagrelor 90 mg BID, clopidogrel 75 mg QD*.

Of the cohort, nine patients had tandem anterior circulation occlusions, two had basilar occlusions, and one patient had an MCA occlusion secondary to severe stenosis. Eleven of the 12 patients had a single intra or extra cranial stent placed immediately after thrombectomy. One patient with severe MCA stenosis had re-occlusion after initial mechanical thrombectomy. Intravenous cangrelor was started prior to the subsequent thrombectomy on this patient, and did not require intracranial stenting.

Patients were kept on intravenous cangrelor for an average of 18.5 h (IQR 995–1,426 min) after which they were transitioned to oral aspirin and ticagrelor (4; 33%) or clopidogrel (8; 67%). P2Y12 levels were assessed in four patients; one while on cangrelor infusion, two after transition to clopidogrel and aspirin, and one after transitioning to ticagrelor and aspirin. Their respective P2Y12 reaction unit levels were 31, 271, 124, and 77. All patients had a repeat neuroimaging within 24 h and seven patients had a third scan after. There were two cases of HT1 per the Heidelberg Bleeding Classification scoring system (9) as assessed on CT brain images, but no other cases of intracranial hemorrhage. Neither patient had worsening intracranial hemorrhage on follow-up scans. There were no cases of in-stent thrombosis, parent vessel thrombosis, or distal thromboembolic events.

Of the studied patients, three (25%) were discharged to home, six (50%) to in-patient rehabilitation, and three (25%) to a skilled nursing facility. Two patients in the cohort died, one of whom had a pulmonary embolism at a skilled nursing facility and another who entered hospice care after discharge at a skilled nursing facility. At 90 day follow-up, all remaining 10 patients had a good functional outcome with a modified Rankin score of ≤ 2 or return to baseline functional status and no recurrent TIA or ischemic stroke (see Supplementary Table 1 for mRS breakdown).

### Comparison of Anterior Circulation Tandem Occlusions With and Without Cangrelor

Prior to the availability of intravenous cangrelor, anterior circulation tandem occlusions were loaded with aspirin (325 mg) and clopidogrel (600 mg) acutely via a nasogastric tube peri-procedurally (no-cangrelor group). Patients with anterior circulation tandem occlusions who received intravenous cangrelor for acute proximal internal carotid artery stenting (*n* = 9) had similar baseline demographics and NIHSS scores to those in the no-cangrelor (dual antiplatelet) group (*n* = 23) (See Table 3). However, the cangrelor group had a higher median ASPECT score (10 vs. 8; *p* = 0.008). Although not significant, cangrelor tandem occlusions had a lower number of ICA terminus occlusions (11 vs. 26%; *p* = 0.40) and shorter procedure times (51 vs. 82 min; *p* = 0.79). Successful reperfusion was comparable between the two groups (100 vs. 96%; *p* = 1) as was a good functional outcome (89 vs. 61%; *p* = 0.21) and mortality (11 vs. 22%; *p* = 0.65). Three patients in the dual antiplatelet group suffered symptomatic ICH and none occurred in the cangrelor group (*p* = 0.54).

**Table 3:** Anterior circulation tandem occlusions treated with acute administration of oral antiplatelets compared with those who were treated acutely with intravenous cangrelor.

	**Total (%)**	**Tandem – Dual Antiplatelets; *n* = 23 (%)**	**Anterior Circulation Tandem – Cangrelor; *n* = 9 (%)**	***P*-value**
**Age** (yrs; mean ± SD)	68.5 ± 13.1	68.8 ± 14.3	67.9 ± 9.8	0.87
**Female**	13 (40.6)	9 (39.1)	4 (44.4)	1.0
**Hypertension**	28 (87.5)	20 (87)	8 (88.9)	1.0
**Diabetes mellitus**	9 (28.1)	5 (21.7)	4 (44.4)	0.23
HbA1c (mean ± SD)	6.3 ± 1.2	6.2 ± 1.2	6.7 ± 1.3	0.30
**Atrial fibrillation**	6 (18.8)	4 (17.4)	2 (22.2)	1.0
**Hyperlipidemia**	23 (71.9)	16 (69.6)	7 (77.8)	1.0
LDL (mean ± SD)	91.2 ± 36.9	94.1 ± 40.2	83.8 ± 27.6	0.49
**Smoking**	13 (40.6)	9 (39.1)	4 (44.4)	1.0
**CAD**	10 (31.3)	6 (26.1)	4 (44.4)	0.41
**NIHSS**	15 (12–18)	15 (11–18)	15 (12–18)	0.56
**IV-tPA**	20 (62.5)	14 (60.9)	6 (66.7)	1.0
**ASPECT score**	9 (7–9)	8 (7–9)	10 (9–10)	0.008
**Door to RP (min)**	102 (67–177)	117 (87–185)	62 (45–111)	0.21
**Onset to RP (min)**	260 (213–406)	312 (236–416)	241 (111–272)	0.12
**Time to follow-up scan (min)**	1,032 (700–1,244)	1,125 (839–1,248)	720 (300–1,080)	0.08
**Procedure time (min)**	70 (50–108)	81.5 (52.5–100)	51 (47–115)	0.79
**Volume of infarct**	7.3 (1.4–23.2)	10.8 ± (2.1–54)	6.6 (0.6–12.9)	0.07
**Location of occlusion**				
MCA	14 (43.8)	9 (39.1)	5 (55.6)	0.79
M2	11 (34.4)	8 (34.8)	3 (33.3)	
ICA-T	7 (21.9)	6 (26.1)	1 (11.1)	
**Successful RC**	31 (96.9)	22 (95.7)	9 (100)	1.0
**Good clinical outcome**	22 (68.8)	14 (60.9)	8 (88.9)	0.21
**Mortality**	6 (18.8)	5 (21.7)	1 (11.1)	0.65
**sICH**	3 (9.4)	3 (13)	0	0.54
**Hemorrhagic transformation score**				
0	21 (65.6)	14 (60.9)	7 (77.8)	0.88
1	7 (21.9)	5 (21.7)	2 (22.2)	
2	0	0	0	
3	1 (3.1)	1 (4.3)	0	
4	3 (9.4)	3 (13)	0	

^*^CAD, coronary artery disease; NIHSS, National Institute of Health Stroke Scale; MCA, middle cerebral artery; M2, M2 division of middle cerebral artery; ICA-T, internal carotid artery terminus; Successful RP, successful reperfusion; sICH, symptomatic intracranial hemorrhage; HT, hemorrhagic transformation score.

^**^Continuous variables represented as mean ± standard deviation or median (interquartile range).

## Discussion

This single center study evaluated 12 patients who received intravenous cangrelor in acute ischemic stroke and found no cases of symptomatic intracranial hemorrhage or in-stent thrombosis. When compared to historical controls with similar occlusions, use of intravenous cangrelor for tandem occlusions found similar rates of good clinical outcome, procedure time, and symptomatic ICH. This study, and others, indicate that acute use of intravenous cangrelor in acute ischemic stroke may be safe and effective and requires further evaluation.

Safety and efficacy of cangrelor has been assessed in patients with cardiac disease. In the CHAMPION PCI study, 8,716 patients with acute coronary syndrome were randomized to receive intravenous cangrelor for a minimum of 2 h followed by transition to clopidogrel, or a clopidogrel 600 mg load at the beginning of the procedure (4). The study was terminated prematurely with 98.6% of planned enrollment completed after an interim analysis revealed that the study was unlikely to show superiority of cangrelor over clopidogrel. Primary endpoints of death, myocardial infarction, revascularization, and in-stent thrombosis were similar between the two groups. Additionally, cangrelor and clopidogrel had similar rates of major bleeding. Following this study, the double-blind placebo-controlled CHAMPION PHOENIX trial compared cangrelor infusion to clopidogrel 300–600 mg load at the start or end of PCI in patients with stable angina, NSTEMI, or STEMI (5). Cangrelor reduced the primary composite end point of death, MI, ischemia-driven revascularization, and death in this study (4.7 vs. 5.9%; CI 0.66–0.93, *p* = 0.005). Intravenous infusion of cangrelor achieved lower rates of intra-procedural (0.6 vs. 1.0%; CI 0.42–0.99, *p* = 0.04), 30-d stent-thrombosis (0.8 vs. 1.4%; CI 0.43–0.9; *p* = 0.01), and procedural complications (3.4 vs. 4.5%; 0.61–0.9; *p* = 0.002) with similar rates of minor and major bleeding. Finally, the BRIDGE study evaluated intravenous cangrelor in a randomized multicenter trial of 210 patients with ACS or post-coronary stenting who were awaiting CABG surgery (10). Compared to placebo, cangrelor infusion at lower titrated doses based on P2Y12 Reaction Units (PRUs) for 2–7 d resulted in higher rates of platelet inhibition. Incidence of bleeding and ischemic events were low in both groups, likely secondary to the lack of power to assess for a difference between the two groups.

Use of cangrelor from cardiology cannot be extrapolated for use in ischemic stroke cases. Patients with acute ischemic stroke, particularly after intravenous tPA or those with low ASPECT scores are at higher risk of intracranial hemorrhage. Furthermore, AIS patients are often not able to follow commands and approximately one fourth of all AIS patients have dysphagia, limiting the use of oral antiplatelets (11). These patients require placement of uncomfortable nasogastric tubes in the neuroangiography suite which often requires sedation in patients not under general anesthesia. Lastly, at the time of acute antiplatelet therapy in AIS, the volume of core infarct is unknown and may create challenges in patients who require early decompressive hemicraniectomy.

Evidence for early stenting for AIS due to tandem occlusions is limited and has not been studied in prospective randomized trials. A meta-analysis of tandem occlusions revealed similar rates of good clinical outcome, procedural complications, and intracranial hemorrhage in patients with stenting or angioplasty of the carotid lesion (12). However, acute stenting treats the symptomatic vessel provoking thrombus and reduces risk of early re-occlusion. Balloon angioplasty alone is not the standard of care for carotid stenosis and may increase the risk of vessel dissection and re-stenosis. Sub-analysis of the STRATIS registry revealed that patients who underwent acute stenting of extracranial carotid stenosis had a higher rate of good clinical outcomes with similar safety to patients not undergoing stenting (13). All nine patients with tandem occlusions had a good functional outcome in our case series, likely a reflection of the short onset to reperfusion times (mean 245 ± 176 min). On the other hand, early stenting with irreversible oral dual antiplatelet therapy may result in poor clinical outcomes if there is any hemorrhagic transformation.

Acute stenting may also be used as a rescue technique after failed mechanical thrombectomy or for severe intracranial stenosis. Although current evidence indicates that stenting for intracranial atherosclerotic disease should be performed at least 7 d after stroke, high grade stenosis with intraprocedural re-development of superimposed thrombus is a subset of AIS patients who require acute stenting. Two of our patients required acute intracranial stents for severely stenotic, re-occluding posterior circulation strokes. Furthermore, use of intravenous antiplatelet therapy may prevent re-development of superimposed thrombus and allow for delayed intracranial stenting as noted in one of our study patients with severe stenosis of the MCA M1 division.

Intravenous cangrelor competitively inhibits the P2Y12 receptor with high affinity and provides a rapid onset of ADP-induced platelet inhibition in the acute stroke setting. Cangrelor does not have any clinically significant drug interactions, does not require activation via hepatic metabolism, and is not affected by renal function, making it an effective weight-based, dose-dependent medication in stroke patients in whom complete medical history may not be available. Its linear pharmacokinetics allows for a dependable amount of platelet inhibition and rapid dephosphorylation by nucleosides allow for rapid inactivation. Lastly, platelet-induced aggregation to the agonist ADP can be tested using VerifyNow Platelet Reaction Units (PRU). Optimal PRU ranges are ambiguous but patients can be assessed to determine their hypo (>240) or hyper-responder (<60) status (14).

While most studies evaluated intravenous cangrelor infusion for short periods, the BRIDGE trial allowed cangrelor infusion for up to 7 d (10). Most patients in our cohort were transitioned to oral antiplatelets within 24 h. Cangrelor is a non-thienopyridine adenosine triphosphate analog and can competitively block binding to the ADP receptor by the unstable clopidogrel metabolite. Hence, oral clopidogrel was administered at the completion of the cangrelor infusion similar to the protocol used in the CHAMPION PHOENIX study (5). Alternatively, the non-competitive ADP analog ticagrelor has a longer half-life and no significant interaction with cangrelor (15). When ticagrelor was used, it was administered approximately half an hour prior to cangrelor infusion cessation. Similarly, aspirin, an irreversible cyclooxygenase 1 and 2 inhibitor, can be administered at any time during the cangrelor infusion as it does not bind to ADP receptors.

Other intravenous antiplatelet medications used in interventional cardiology have limited studies in neurointervention. Glycoprotein 2b/3a inhibitors function downstream of ADP analogs and inhibit the final pathway of platelet aggregation by preventing fibrinogen binding to platelets and cross-linking to other platelets. While early clinical trials showed benefit of these agents in reducing cardiovascular events, use of these agents also resulted in more bleeding (16). Use of potent dual antiplatelet regimens, second generation stent technologies, and other advances in interventional cardiology has limited the use of Gp 2b/3a inhibitors to patients with a large thrombus burden or as a bail out therapy in high risk PCI interventions (17).

Given the increased risk of bleeding with Gp2b/3a inhibitors, use of these agents has been limited in acute ischemic stroke studies. Abciximab has been shown to be associated with significant increases in symptomatic intracranial hemorrhage without any improvement in long-term functional outcomes (18). However, half-dose abciximab bolus without infusion for acute stenting in stroke was shown to be safe in 99 patients (19). Eptifibatide in conjunction with intravenous tPA has been shown to be safe in a small clinical trials of 27 patients (20). A recent study in 29 patients who underwent acute carotid stenting for tandem occlusions found that eptifibatide was associated with low rates of symptomatic intracranial hemorrhage (21). Similarly, tirofiban has been shown to be safe in acute ischemic stroke (22) and in emergent carotid stenting (23). However, Gp2b/3a inhibitors are associated with spontaneous pulmonary hemorrhage in up to 1% of patients which are likely under-reported and can be fatal in up to 50% of cases (24–26). Lastly, Gp2b/3a inhibitors may affect PRU results which can make interpretation of antiplatelet activity challenging in the early stroke period (27).

Intravenous cangrelor provides a useful additional tool for managing acute ischemic strokes. Preliminary studies of the use of cangrelor for stenting in acute ischemic stroke has shown it be a promising and feasible alternative to oral antiplatelet therapies (see Table 4). In a study by Linfante et al., full cardiac dosing of cangrelor was used in five ischemic stroke patients and resulted in gastrointestinal bleeding in one patient (33). A French study with a similar cangrelor dosing regimen resulted in one case of symptomatic intracranial hemorrhage, two cases of asymptomatic intracranial hemorrhage, and one retroperitoneal bleed amongst 12 stroke patients (31). The largest case series published from Italy found no cases of peri-procedural in-stent thrombosis in 38 AIS patients requiring acute stenting and three cases of delayed in-stent thrombosis when cangrelor was transitioned to an antiplatelet other than ticagrelor. In this study, Cervo et al. also used the full cardiac dose of cangrelor and had four patients who developed basal ganglia intracranial hemorrhages requiring cangrelor discontinuation, although it was deemed safe to continue in two patients with post-procedure asymptomatic intracranial hemorrhage (30). When half the cardiac dose was used, similar to our study, all seven patients with acute ischemic stroke had adequate (PRU < 200) antiplatelet activity and no evidence of intracranial hemorrhage or in-stent thrombosis (29). Lastly, a study by Entezami et al. treated 21 patients with ischemic stroke or symptomatic critical stenosis (32). A cangrelor bolus of 5 mcg/kg was followed by a maintenance infusion of 0.75 or 1 mcg/kg/min. Patients in this study had cangrelor infusion rates adjusted based on post-procedure PRU levels. One patient required up-titration and two required a lower dose of cangrelor. While none of the ischemic stroke patients had in-stent thrombosis, 2 out of 37 patients in the study did. Our preliminary experience, combined with previous clinical studies, suggest that a half cardiac dose of cangrelor is safe and efficacious (8). Due to its pharmacokinetics, intravenous cangrelor allows neurointerventionalists and neurosurgeons to strictly control antiplatelet activity in the acute stroke setting and allows for rapid changes in management according to a patient's clinical status.

**Table 4:** Summary of recently published studies evaluating intravenous cangrelor for cerebrovascular diseases.

**Study**	**Sample size (n)**	**Study design, included patients (country)**	**Endpoints**	**Antiplatelet dosing**	**Results**
Abdennour et al. (28)	7	Retrospective, observational case series in: SAH & unruptured aneurysms (France)	Treatment-related complications (minor/major hemorrhagic complications, ischemic complications) Clinical and angiographic outcomes (average 9 months)	Cangrelor 30 μg/kg bolus of + IV Aspirin 250 mg Cangrelor IV infusion 4 μg/kg/min for 12–48 h. Transition to ticagrelor 180 mg + ASA 81 mg	One patient (14%) had worsening of ICH followed by hemorrhagic transformation of ischemic stroke resulting in death One asymptomatic ICH (both ICHs contributed to by ticagrelor) No in-stent thrombosis No mRS worsening observed at discharge, (except for the patient who died); six out of seven patients had a mRS ≤ 2 at follow-up
Aguilar-Salinas et al. (29)	8	Retrospective, observational case series in: AIS & unruptured aneurysm (USA)	Intra-procedural thromboembolic complications or in-stent thrombosis Composite of ischemic stroke or ICH at 24 h Composite primary outcome at 7 d and 30 d, in-stent thrombosis at 48 h after the intervention, TIA within 30 d, and all-cause mortality rate	Cangrelor 15 μg/kg bolus of + Aspirin 325 mg Cangrelor IV infusion 2 μg/kg/min for > 2 h or until procedure conclusion Transition to ticagrelor 180 mg	No intra-procedural thromboembolic complications, intra-procedural in-stent stenosis, or stroke No in-stent thrombosis at 48 h after the intervention, TIAs or death within 30 d
Cervo et al. (30)	38	Retrospective, observational case series in: AIS requiring intra or extracranial stenting (Italy)	In-stent thrombosis, intracranial hemorrhage requiring cangrelor discontinuation	IV aspirin 500 mg followed by cangrelor 30 μg/kg bolus followed by 4 μg/kg/min Transition to ticagrelor 180 mg + ASA 100 mg	Four cases of post-procedure in-stent thrombosis: Three within 24 h (7.9%): two were after cangrelor discontinuation & switched to clopidogrel instead of ticagrelor; the third had an in-stent occlusion from proximal clot propagation One delayed in-stent thrombosis (<7 days): After switching ticagrelor to ticlopidine Two SAH post-procedure, continued on cangrelor Four basal ganglia ICH – stopped cangrelor, one patient required surgical evacuation
Elhorany et al. (31)	12	Retrospective, observational case series in: AIS (France)	Intra-procedural thromboembolic complications & in-stent thrombosis. Post-procedural in-stent thrombosis, symptomatic & asymptomatic ICH, acute ischemic stroke within 48 h, groin puncture complications requiring surgical repair or blood transfusion, TIA, recurrent AIS and the all-cause mortality rate within 3 months	Cangrelor 30 μg/kg bolus of + IV Aspirin 250 mg Cangrelor 4 μg/kg/min for 12–48 h. Transition to ticagrelor 180 mg + ASA 81 mg	No intra-procedural thromboembolic complications Post-procedural in-stent thrombosis after cangrelor cessation for emergent craniotomy for malignant infarction (8%) Two (17%) asymptomatic ICH, one (8%) symptomatic ICH, and one (8%) retroperitoneal hematoma
Entezami et al. (32)	37	Retrospective observational case series in: AIS, symptomatic stenosis, SAH, unruptured aneurysms, carotid-cavernous fistula, carotid blowout, and pseudoaneurysm (USA)	In-stent thrombosis and hemorrhagic complications	Cangrelor 5 μg/kg bolus followed by infusion of 0.75–1 μg/kg/min	One (2.7%) intraprocedural occlusion, one (2.7%) patient post-procedure in-stent occlusion No hemorrhagic complications
Linfante et al. (33)	10	Retrospective, observational case series in: AIS, SAH, and unruptured aneurysm (USA)	Peri-procedural complications including bleeding and thrombotic events Discharge disposition and 90 d mRS	Cangrelor 30 μg/kg bolus followed by 4 μg/kg/min for 2 h Transition to ticagrelor 180 mg + ASA 81 mg	Asymptomatic SDH in one case (10%), one case (10%) of worsening of IVH (died) One transient GI bleed post-procedure (10%)

^*^AIS, Acute ischemic stroke; SAH, subarachnoid hemorrhage.

While our study did not evaluate the cost implications of a prolonged cangrelor infusion, sub-analysis of the CHAMPION PHOENIX study revealed that the increased cost of cangrelor may be offset by a reduction in intra-procedural thrombotic events during PCI resulting in lower major adverse cardiac events, mortality, procedural and hospitalization costs, and shorter hospital stays (6). Rates of major and minor bleeding were similar between cangrelor and clopidogrel in this study, but needs further study in the ischemic stroke population who are prone to intracranial hemorrhage. Ultimately, larger studies are needed to determine the cost-effectiveness of intravenous cangrelor in AIS patients.

### Limitations

This study has multiple limitations. This is an underpowered single-center study and results may not be generalizable to other centers. Furthermore, patients included in the study had a range of intracranial occlusions. While we compared anterior circulation tandem occlusions with and without cangrelor, this study did not provide sufficient power to detect large differences between the therapies. Lastly, antiplatelet effect was not assessed by P2Y12 levels, different stents were used during neurointerventional procedures, and patients were transitioned off cangrelor to different oral antiplatelet therapies at different doses.

## Conclusion

Intravenous cangrelor is a promising medication for patients with acute ischemic stroke who require an immediate antiplatelet effect. Larger prospective studies on the efficacy, safety, and effect on procedure times of intravenous cangrelor in neurointervention are warranted.

## Data Availability Statement

The original contributions presented in the study are included in the article/Supplementary Material, further inquiries can be directed to the corresponding author/s.

## Ethics Statement

The studies involving human participants were reviewed and approved by Promedica Neurosciences Insitutional Review Board. Written informed consent for participation was not required for this study in accordance with the national legislation and the institutional requirements.

## Author Contributions

HS contributed to the conception of the work, data collection, data analysis and interpretation, and drafting the article. GD contributed to the data collection and drafting of the article. SZ contributed to the conception of the work and critical revision of the article. JS contributed to the data collection of the article. RB contributed to the critical revision of the article. MJ contributed to the conception of the work, data interpretation, critical revision of the article, and final approval of the version to be published. All authors contributed to the article and approved the submitted version.

## Conflict of Interest

The authors declare that the research was conducted in the absence of any commercial or financial relationships that could be construed as a potential conflict of interest.
