# Supplementary material for: Safety of Low Dose Intravenous Cangrelor in Acute Ischemic Stroke: A Case Series
Source: Front Neurol. 2021 Jun 4;12:636682. doi: 10.3389/fneur.2021.636682 (PMC8211882; doi:10.3389/fneur.2021.636682)
Supplement: Supplementary file 1 [file Table_1.DOCX]

**SUPPLEMENTARY MATERIAL:**

**Supplemental Table 1:** mRS breakdown of patients receiving intravenous cangrelor for acute ischemic stroke

| **mRS Score** | **Cangrelor** |
| --- | --- |
| 0 | 2 (16.7) |
| 1 | 3 (25) |
| 2 | 4 (33.3) |
| 3 | 1 (8.3)* |
| 4 | 0 |
| 5 | 0 |
| 6 | 2 (16.7) |
| *Baseline mRS = 3 | |
